# Twelve-year trend of Escherichia coli antibiotic resistance in the Islamabad population

**DOI:** 10.1016/j.amsu.2022.103855

**Published:** 2022-05-27

**Authors:** Kashif Bangash, Hassan Mumtaz, Mehwish Mehmood, Majid Ali Hingoro, Zoobia Z. Khan, Ahmed Sohail, Sami Ullah, Durishahwar Maqbool, Umm-E-Farwa, Neelum Jamal, Momina Sami Khan, Shahzaib Ahmad, Anum Sohail, Hassan ul Hussain, Irfan Ullah

**Affiliations:** a KRL Hospital Islamabad Pakistan, Pakistan; b Maroof International Hospital. Public Health Scholar, Health Services Academy, Islamabad, Pakistan; c Mohiudin islamic Medical College AJ & K, Pakistan; d Clinical Research Center, Shifa International Hospital Islamabad, Pakistan; e GDMO, PNS Hafeez, Islamabad, Pakistan; f Histopathologist, Fauji Foundation Hospital Rawalpindi, Pakistan; g Department of Community Medicine. Fazaia Ruth Pfau Medical College (FRPMC) Karachi, Pakistan; h Shaheed Benazir Bhutto Women's University Peshawar, Pakistan; i King Edward Medical University Lahore, Pakistan; j Dow University of Health Sciences, Karachi, Pakistan; k Kabir Medical College, Gandhara University, Peshawar, Pakistan

**Keywords:** E. coli, Antimicrobial susceptibility, Uropathogenic, UTI, Trends, Lifestyle medicine

## Abstract

**Objective:**

Increasing rates of antimicrobial resistance among E. coli is a growing concern worldwide. We aimed to assess the changing antibiotic sensitivity pattern in Uropathogenic E. coli over a period of 12 years with special emphasis on ESBL-producing E. coli.

**Methods:**

A retrospective study was done on the population of Islamabad from 1st Jan 2005 to Dec 2010 and then continued from 1st Jan 2016 to 31st May 2021. A total of 12000 samples were analyzed for isolation and identification of bacteria and antimicrobial susceptibility testing, from patients having uncomplicated urinary tract infections. Our primary was to find the antibiotics with the highest sensitivity against E. Coli in 2021, while our secondary outcome was to find the overall sensitivity pattern of E. Coli to antibiotics from 2005 to 2021.

**Results:**

There was a decrease in susceptibility rates of E. coli to Amoxicillin-Clavulanic Acid 47%, Trimethoprim-Sulfamethoxazole (TMP-SMX) 27%, Fluoroquinolones 24% & Cephalosporins 38%. There was a significant increase in the use of Nitrofurantoin and Fosfomycin. High resistance rates to Fluoroquinolones (76%), TMP-SMX (73%), Cephalosporins (62%), and Amoxicillin (53%) were documented. However, significantly high degree of sensitivity rates to Fosfomycin (92%), Aminoglycosides (90%) & Nitrofurantoin (80%) were recorded.

**Conclusions:**

Uropathogenic E. coli shows the highest sensitivity to Fosfomycin and Aminoglycosides in the year 2021, followed by Nitrofurantoin and Sulbactam. Cephalosporins, Amoxicillin/Cluvalanic acid, TMP-SMX, and Fluoroquinolones show a declining sensitivity pattern. UTIs can be prevented well by lifestyle changes, taking vitamins, trace elements, and carbohydrates.

## Highlights

• This article is discussing the antibiotic sensitivity pattern in Uro-pathogenic E. coli over a period of 12 years having a special emphasis on ESBL-producing E. coli
• Uropathogenic Ecoli have the highest sensitivity to Fosfomycin and Aminoglycosides in the year 2021, followed by nitrofurantoin and Sulbactam.
• Dietary recommendations could be a first step toward prevention of UTI recurrence because eating habits appear to be a key risk factor.
• UTIs can be prevented well by taking vitamins, trace elements, and/or carbohydrates. These antibacterial agents work well when combined. Antibiotic overuse and the prevalence of antibiotic-resistant microbes pose a serious danger to the use of probiotics.
• Further monitoring of antimicrobial susceptibility is recommended in the upcoming years for effective treatment.

## 1 Introduction

There is currently a worldwide epidemic of recurring and chronic urinary tract infections (UTIs), making them one of the most common infectious diseases. The most common cause of UTIs worldwide is Escherichia coli (E. coli). Up to 80% of UTIs are caused by the bacteria E. coli [1].

Being one of the most prevalent bacterial infections globally, UTIs are more frequently reported in women as compared to men due to lesser distance to the bladder in women. The urethral opening being closer to the rectum in women increases the chances of frequent UTIs in women [2]. Acute UTIs are categorized into cystitis (Lower UTIs) and pyelonephritis (Upper UTIs) [1].

UTIs can also be clinically classified into complicated and uncomplicated UTIs [3] and are recognized as one of the most common childhood bacterial infections [4] and the reason for being absent from the workplace [5]. Bacteria are preponderant amongst all the etiologies of UTIs, E. coli being the on top followed by Staphylococcus saprophyticus species [6].

Antimicrobial empiric treatment is given when symptoms of lower UTI like suprapubic pain, urinary incontinence, dysuria, and frequent urination are present [7,8]. A speedy increase has been noticed in antibiotic resistance worldwide, especially in the last ten years [9]. Extended-spectrum beta-lactamase (ESBL) producing strains constitute a majority of E. coli infections, which are resistant to extended-spectrum antibiotics such as cephalosporins and monobactams [10,11]. ESBL is an enzyme found in ESBL-producing bacteria which assists them in resisting certain antibiotics [12].

Enzymes including SHV, CTX-M, and TEM encoded by the genes in plasmids play a pivotal role in mediating ESBL (13). Surprisingly, the predominance of ESBL-producing E. coli is rising in Europe [14], Asia [15], Africa [16], and Mediterranean regions [17,18] in spite of geographical and racial differences.

UTI treatments depend on numerous factors such as infection type, allergic history, age, gender, and pattern of pathogen's antibiotic susceptibility [[19], [20], [21]]. Antibiotics namely β-lactams, Trimethoprim, and Nitrofurantoin are preferred for treating uncomplicated UTIs [19], while Fluoroquinolones are widely used for treating both complicated and uncomplicated UTIs in some countries [22].

Risk factors include long-term hospitalization, use of catheters, and use of antibiotics prior to UTI can lead to multi-drug resistance [19,20]. The predominance of multi-drug resistant strains has been reported to be more than 65%, since 2011, especially in Pakistan [23,24]. Excessive use of Quinolones and Fluoroquinolones has resulted in raised resistance in UTI-causing bacteria [25].

Despite being a threatening concern, inadequate research has been conducted on this topic in a third-world country like Pakistan. There was a dire need to study the sensitivity patterns in Uropathogenic E. coli so that the findings can be used to perform the best possible antibiotic treatment against UTIs.

The primary aim of this study is to assess the altering antibiotic sensitivity patterns in Uropathogenic E. coli from January 2005 – to May 2021, especially focusing on ESBL-producing E. coli.

## 2 Materials and methods

A retrospective study was conducted on the population of Islamabad presenting with UTIs in the tertiary care centers. Islamabad is Pakistan's ninth-largest city, with a population of approximately one million one hundred sixty-four thousand. According to the Globalization and World Cities Research Network, Islamabad is a Gamma + city. The city has an urban area of 220.15 km^2^ (85.00 sq mi) and a metro area of 1385.5 km^2^ (534.9 sq mi). It has a density of 2089 inhabitants per square kilometer (5410 inhabitants per square mile) [26]. Our study is fully compliant with the STROCSS 2021 guidelines [27]. A complete STROCSS 2021 checklist has been provided as a supplementary file. Our study has been registered on Research Registry with the following UIN: researchregistry7926 [28]. Our study is in accordance with the Declaration of Helsinki.

### 2.1 Inclusion criteria

Urine culture samples were collected from patients having uncomplicated UTIs who presented with at least three of the following symptoms: dysuria, urgency, frequency, or suprapubic tenderness and had a positive urine culture for E. coli (10*5 CFU/mL).

### 2.2 Exclusion criteria

Urine samples that were culture-negative, had no significant growth or had pathogens other than E. Coli were excluded.

### 2.3 Data collection

The study was conducted in two phases, one from 1st Jan 2005 to Dec 2010 then phase two continued from 1st Jan 2016 to 31st May 2021.

Data was collected after the ethical approval was obtained from KRL Hospital Islamabad, wide letter no “**Ref ERC: KRL-HI-ERC/Apr17/18–5**”

A total of 12000 samples were analyzed for isolation and identification of bacteria and antimicrobial susceptibility testing.

### 2.4 Identification of uro pathogens & susceptibility testing to antibiotics

Antibiograms for the bacteria were obtained to test the sensitivity of the antibiotics. Bacterial growth was considered positive on the dipslide at 10*5 CFU/mL. Two or more bacterial species growing together were considered polluted and were eliminated. The isolated bacteria were kept at a temperature of 20 °C in a solution of peptone/glycerol (30% w/v).

Amoxicillin/Clavulanic acid (625mg/1 gm), Trimethoprim/Sulphamethoxazole (TMP-SMX) (160/800 mg), Aminoglycosides (80mg/500 mg), Nitrofurantoin (3 gm), Cephalosporins (400 mg), Sulbactams (1 gm/2 gm), and Fosfomycin (100 mg) were tested.

### 2.5 Primary outcome

To find the antibiotics with highest sensitivity against E. Coli in 2021.

### 2.6 Secondary outcome

To find the overall sensitivity pattern of E. Coli to antibiotics from 2005 to 2021.

The Approval by the Ethics Committee & Consent to Participate:

The ethical review committee of KRL Hospital Islamabad accepted this study in a letter titled “Ref ERC: KRL-HI-ERC/Apr17/18–5". The informed consent from the patients was obtained considering Helsinki's Declaration.

### 2.7 Statistics

IBM SPSS version 24 was used to compile and analyze the data. A p-value of <0.05 was considered as significant.

### 2.8 Results

**Fig. 1:**
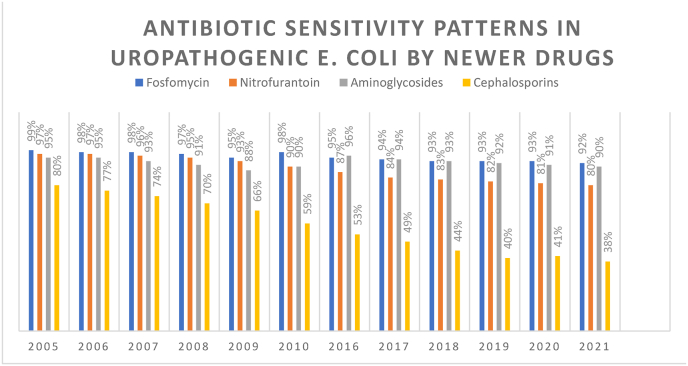
Antibiotic sensitivity patterns in newer drugs.

Cephalosporins had a similar trend of decreasing sensitivities. The sensitivity for cephalosporins was 80% in 2005, after 11 years, it decreased to 53% in 2016, and after 5 years, it further declined to 38% (Fig. 1).

**Fig. 2:**
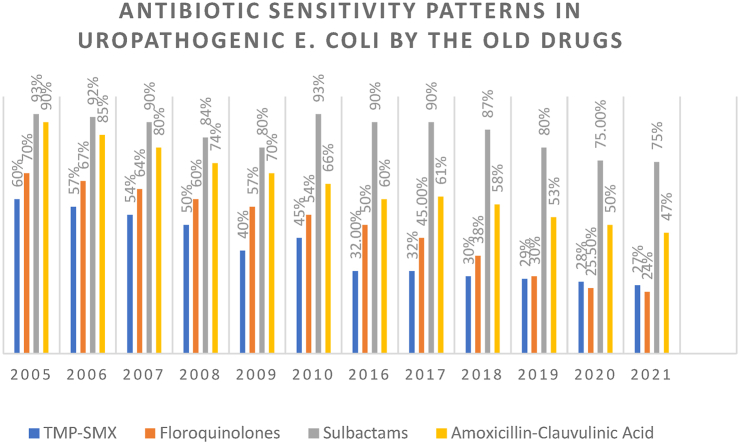
Antibiotic sensitivity patterns in older drugs.

The sensitivity pattern of older drugs has been illustrated in Fig. 2. Amoxicillin in conjunction with Clavulanic acid showed a progressive decrease in sensitivity over the years starting from 90% in 2005 to a mere 47% in 2021.

Hence, both beta-lactam antibiotic groups suffered a declining trend in sensitivities against UTI-causing E. coli. Whereas Fosfomycin was found to be effective against UTI-causing E. coli. In 2005. It was the most effective antibiotic against UTI-causing E. coli with a sensitivity value of 99%.

Over the course of 16 years, the sensitivity value decreased by only 7% and in 2021, Fosfomycin is sensitive against 92% of the UTI-causing E. coli bacteria.

**Fig. 3:**
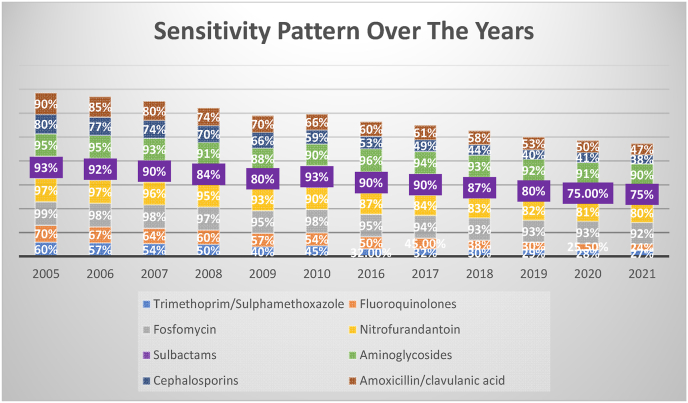
Sensitivity patterns of various antibiotics from 2006 to 2021.

In 2005, aminoglycosides were highly effective (sensitivity level 95%) like Fosfomycin. In 2021, the sensitivity level was 90%. Fluoroquinolones and TMP-SMX had lower sensitivities against UTI-causing E. coli in 2005 (70% and 60%, respectively) and a progressive decline in sensitivity values was observed. In 2021, the sensitivity values of Fluoroquinolones and TMP-SMX were 24% and 27%, respectively (Fig. 3).

## 3 Discussion

UTI is the most common cause of antibiotic prescription in primary care setup [1,2]. Women are more prone to urinary tract infections due to shorter urethra and anatomic differences. E. coli is the comment cause of UTIs, and accounts for 80% of uncomplicated UTIs [3,4].

The resistance of TMP and SXT to TMP-SMX has increased over 10 years. This was in contrast with another study conducted in France in 2018 that reported 78% and 75% susceptibility of these drugs to E. coli respectively [5,6].

Another study conducted in 2013 found 26.6% resistance to trimethoprim [7]. This difference in resistance rate to TMP can be explained by the different settings in which the study was conducted. A study found the use of TMP as a single drug has increased its resistance [8]. Increase resistance of TMP/SMX to E. coli over a decade in our region as compared to developed countries do not recommend its use to treat uncomplicated UTI in developing countries.

Fosfomycin is suggested to be a safe and effective drug in treating UTIs. Of the E. coli isolates studied, a decade-long study showed a declining trend in sensitivity from 98%, a decade ago to 95% in 2016 to 93% in 2021 [9,10].

Piperacillin/Tazobactam (PIP-TAZ) can be as effective in treating community-acquired uncomplicated ESBL producing strains of E. coli as trimethoprim, a study has shown. A study from Korea revealed 87.3% susceptibility of PIP-TAZ to E. coli in 2017 as compared to 98.9% in 2008 [11].

The susceptibility of Fluoroquinolones continuously decreased from 60% in 2005–2010 to 25% in 2017 and then increase to 38%. Hence there is a significant decrease over a period of 10 years. There was a continuous decline in susceptibility during the study period and it remained less than 70% throughout the study period. The susceptibility rate of Fluoroquinolones was even less than TMP-SMX over a period of 10 years. A study reported markedly increased resistance to Fluoroquinolones from 2008 to 2017 (79.5% vs 58.6%) [11].

Another study found increased resistance to both Fluoroquinolones and Trimethoprim [14]. The increase in resistance over the years can be explained by the non-judicious use of Quinolones in developing countries [12].

Our study reported more than 90% susceptibility of Aminoglycosides over a period of 10 years. This contradicted the findings of a previous study that reported less than 80% susceptibility of ESBL-producing strains of E. coli to Gentamicin. A study conducted in 2014 in New Delhi found 46.7% resistance to Gentamicin [13]. This finding is not consistent with our study where we reported maximum antibiotic sensitivity with Aminoglycosides. Regional differences account for differences in the sensitivity pattern of antibiotics.

Our study corroborates the findings of a previous study conducted in Poland from 2016 to 2018 that found maximum susceptibility of Amikacin followed by Piperacillin and Gentamicin in managing UTIs. Aminoglycosides are not frequently prescribed drugs due to their nephrotoxicity. With regards to oral antibiotics, the highest sensitivity rate was observed with Fosfomycin and nitrofurantoin [14].

Our study reported E. coli showing 27% susceptibility to 3rd generation Cephalosporins, particularly Ceftriaxone. This has decreased over a decade from 69%. The high susceptibility pattern of Nitrofurantoin is explained by the fact that the drug is not frequently available [15].

In our study, most ESBL-producing strains of E. coli are resistant to Trimethoprim, Fluoroquinolones, Amoxicillin/Clavulanic acid, and Ceftriaxone. The antibiotic susceptibility pattern shows maximum resistance with ceftriaxone, followed by Trimethoprim, Fluoroquinolones, and Amoxicillin/Clavulanic acid. These are the drugs most frequently prescribed by general physicians.

The ESBL-producing E. coli shows maximum sensitivity with Aminoglycosides, followed by Fosfomycin, PIP-TAZ, and Nitrofurantoin. Our study demonstrated a continuous increase in resistance in frequently used antibiotics.

### 3.1 Lifestyle changes

Winchester hospital claims that UTI treatment requires lifestyle changes that can help flush the bacteria and antibiotics from your urinary system by drinking a lot of water. Taking a toilet break is necessary according to them. If you have intercourse, you should drink a full glass of water and then pass pee. every day you should wash your genitals After a bowel movement, ladies should wipe from front to back and avoid using douches and feminine sprays [29].

Dietary recommendations could be the first step towards the prevention of UTI recurrence because eating habits appear to be a key risk factor [30]. Various research advised various preventative methods, such as drinking cranberry juice (urinary alkalization), over-the-counter cystitis treatment medications, or following certain hygiene habits, such as wiping the genitals from front to back [31]. In order to alleviate symptoms and enhance overall health, natural therapies have been widely employed in the treatment of numerous ailments. First aid and short-term prevention are both possible with herbal remedies. UTIs can be prevented well by taking vitamins, trace elements, and/or carbohydrates. These antibacterial agents work well when combined. Antibiotic overuse and the prevalence of antibiotic-resistant microbes pose a serious danger to the use of probiotics [32].

### 3.2 Strengths and limitations

This study covers antibiotic susceptibility patterns over a period of 12 years. Our results are not generalized as our study only deals with uncomplicated UTIs and not all the patients having UTIs. Huge surveys need to be conducted on a mass level to know the exact declining sensitivity patterns.

## 4 Conclusion

Uropathogenic E. coli shows the highest sensitivity to Fosfomycin and Aminoglycosides in the year 2021, followed by Nitrofurantoin and Sulbactam. Cephalosporins, Amoxicillin/Clavulanic acid, TMP-SMX, and Fluoroquinolones show a declining sensitivity pattern. Further monitoring of antimicrobial susceptibility is recommended in the upcoming years for effective treatment. UTIs can be prevented well by lifestyle changes by taking vitamins, trace elements, and carbohydrates.

## Sources of funding

Nill.

## Ethical approval

Ethical approval was granted by KRL Hospital Islamabad Pakistan. Bearing “**Ref ERC:KRL-HI-ERC/Apr17/18–5”.**

However no patients were involved during the study.

## Consent

“. The informed consent from the patients was obtained considering Helsinki's Declaration.

## Authors contribution

1. The main concept was determined by Kashif Bangash, Durishahwar Maqbool

2. The article is reviewed and approved by Neelum Jamal, Majid Ali Hingoro

3. Collection of data is done by Umme-e-Farwa, Sami Ullah, Ahmed Sohail

1. Data is interpreted by Shahzaib Ahmad, Anum Sohail, Hassan-ul-Hussnain

2. Writing of the manuscript is done by Mehwish Mehmood, Zoobia Z Khan

3. Statistical analysis is done by Irfan Ullah, Momina Sami khan

4. Manuscript editing is done by Hassan Mumtaz

## Registration of research studies

1. Name of the registry:

2. Unique Identifying number or registration ID:

3. Hyperlink to your specific registration (must be publicly accessible and will be checked):

(Trial registry not needed as it does not contain active human participants. Data was collected from hospital records)

## Guarantor

Kashif Bangash & Hassan Mumtaz.

## Provenance and peer review

Not commissioned, externally peer-reviewed.

## Declaration of competing interest

Nill.
